# Decomposition dynamics and nutrient release of walnut orchard litter in the Taihang Mountains, China

**DOI:** 10.1038/s41598-026-40404-x

**Published:** 2026-02-24

**Authors:** Xingrui Zhang, Dan Li, Liying Chen, Haoan Luan, Guohui Qi

**Affiliations:** College of Forestry, Hebei Agricultural University, Baoding, Hebei Province China

**Keywords:** Taihang Mountains, Walnut plantation, Litter, Decomposing features, Ecology, Ecology, Environmental sciences, Plant sciences

## Abstract

The decomposition of walnut litter components (leaves, husks, and male inflorescences) were studied to understand the decomposition process and its role in the soil fertility in walnut plantation in the southern Taihang Mountains of Hebei Province, China. The experiment was carried out at a walnut plantation in the southern Taihang Mountains of Hebei Province, China. The analysis focused on the dynamic changes of four mineral elements, trace elements, and lignin. The results showed that during the 300-day decomposition period, the mass residue percentages of the three types of litter were leaves (64.92%), husks (37.90%), and male inflorescences (21.33%). The 120th day was a turning point in the decomposition process. Before this, the decomposition process was active and relatively rapid; after reaching this inflection point, the rate of decomposition tended to stabilize. The Olson model was used to simulate and predict the decomposition process of three types of litter. The order of the decomposition coefficient *k* for litter types was male inflorescence > husks > leaves. The higher the *k* value, the greater the decomposition rate. The 50% decomposition time estimates for leaves, husks, and male inflorescences were 1.28a, 0.53a, and 0.39a, respectively, with values of 5.54a, 2.28a, and 1.68a for 95% decomposition, respectively. After 300 days, according to the criterion of NR < 100%, the C, N, P, K, and lignin contents of the three types of litter showed a net release state, with a wave release decomposition form and a small enrichment phenomenon occurring during the process. Compared with the initial state of decomposition, the four trace elements generally exhibited a net release state.

## Introduction

Litter, also known as organic debris, is a collective term for all organic matter that is composed of dead aboveground plant components. Litter forms a material and energy resource for decomposers, thereby serving to maintain ecosystem functions^1^. Litter is an important component of forest ecosystems that is closely related to forest stand dynamics, and its decomposition process is the main pathway for nutrient elements to return to the soil^2^. At the same time, litter is also the main source of food for surface and underground decomposers and thus forms important links in the material cycle and energy flow of forest ecosystems^3^. The decomposition rate follows a certain temporal pattern, usually in the form of a single peak or a double peak^4^. On a global scale, many researchers have conducted extensive research on the decomposition rates, characteristics, and nutrient element return patterns of various types of litter under different climate regions, forest types, and land use conditions, and have achieved many important research results^5–10^. Litter is one of the main sources of organic matter in forest soil, and its decomposition is an important process in the biogeochemical cycling of nutrients in forest ecosystems. It is also one of the determining factors in the effective nutrient supply capacity of soil and is closely related to forest productivity. Nutrient element migration model of forest litter during decomposition process. The formula mainly includes: leaching enrichment release mode, enrichment release mode, and direct release mode. The release characteristics of different nutrient elements are different. At present, research on the decomposition of forest litter focuses on exploring influencing factors and distribution patterns, while there are few reports on the dynamic changes in the decomposition process and nutrient cycling mechanisms^11^. Therefore, conducting in-depth research on this complex process is crucial for the efficient and rational management of forest resources.

Walnut (*Juglans regia* L.), also known as Qiangtao, is a deciduous tree in the family Juglandaceae. Walnut is distributed in 53 countries and regions on six continents. Walnuts have a long history of cultivation in China. According to FAO (Food and Agriculture Organization of the United Nations) data, China’s walnut harvest area and yield in 2020 accounted for 31.32% and 33.09% respectively, of the global crop. According to the “China Statistical Yearbook 2022” released by the National Bureau of Statistics, the total walnut production in 2021 was 5.4035 million tons, a year-on-year increase of 12.67%. Hebei Province produced 182,200 tons, accounting for 3.37%, ranking sixth among the major production areas in China. In recent years, researchers have conducted in-depth studies on the selection of superior varieties, optimization of cultivation techniques, interpretation of physiological and ecological characteristics, and improvement of agronomic management measures. However, the walnut forest ecosystem faces problems such as declines in soil fertility, decreased productivity per unit area, and insufficient measures for nutrient element supplementation and allocation. Most of the existing research on the decomposition characteristics of litter has focused on timber forests, ecological protection forests, and other types of fields. However, there are very few research reports concerning litter in forests of economically important crops such as walnuts. Therefore, in this study, we selected three types of litter in a walnut artificial forest located in the Taihang Mountains of Hebei Province and conducted a systematic study on their decomposition dynamics. The aim was to elucidate the pattern of nutrient element return in walnut artificial forests. The results can provide a basis for maximizing the economic and ecological benefits of walnut orchards.

## Materials and methods

### Study site

The experimental area was selected within the Walnut Demonstration Park of Hebei Lvling Fruit Industry Co., Ltd., in Lincheng County, Xingtai City, Hebei Province, China (114° 30’–114° 33’ E and 37° 29’–37° 32’ N). This region has a warm temperate continental monsoon climate, with an average annual temperature of 13 °C, an average annual sunshine time of 2653 h, a frost-free period of 202 days, and an average annual precipitation of 521 mm. Precipitation is concentrated in the summer. The soil type is brown soil, with a soil layer thickness of 40–100 cm. The area has mostly gentle slopes below 10°, and the height range of the poster is 85–135 m. The main variety of walnuts in the park is “Green Ridge,” and planting is largely in a north–south direction, with a dwarfing and dense planting mode. Walnut trees were sampled and planted in 2006, with an age of 13 years (sampled in 2019). The average tree height is 3.5 m.

### Experimental design

The experimental materials were three common types of litter from “Green Ridge” walnuts, namely leaves, green husks, and male inflorescences. The field simulation decomposition of litter adopted the net bag method. The three types of walnut litter were collected from August 2019 to April 2020 and stored for future use. Subsequently, 30.0 g of leaves and male inflorescences and 70.0 g of husks were placed into nylon mesh bags with a specification of 100 mesh. The mesh bags were labeled with the type and placement time, and 36 samples were collected for each type of litter. The samples to be decomposed were placed on the ground below randomly selected walnut tree crowns, and existing undecomposed litter and other debris were removed to ensure that the decomposition bags were in full contact with the ground. The mesh bags were fixed in place with a wire clamp.

The decomposition starting point was May 16, 2020, and thereafter three samples of each litter type were retrieved every 60 days over a period of 300 days. To retrieve the samples, any soil or other debris attached to the mesh bag was removed, and the contents were dried at 85 °C to a constant weight. The dry weight of the residue was recorded, and the content was crushed and sieved, then placed in a self-sealing bag. The lignin, N, P, K, Cu, Zn, Fe, Mn, and organic carbon contents were measured.

### Determination of the chemical properties of litter

The lignin content was determined using the acetyl bromide method. The organic carbon content was determined by the potassium dichromate dilution heat method. The leaves, husks, and male inflorescences were boiled in concentrated sulfuric acid hydrogen peroxide until clear and then used for the measurements. The nitrogen content was determined using a Kjeldahl nitrogen analyzer. The P content was determined using the molybdenum antimony scandium colorimetric method with a UV spectrophotometer. The contents of Cu, Zn, Fe, and Mn were determined by concentrated sulfuric acid hydrogen peroxide digestion followed by measurement using an acetylene air flame atomic absorption spectrophotometer^12^.

### Data processing

The residual rate of litter quality and the Olson model decomposition index were calculated using Microsoft Excel (Microsoft Corp., USA). Analysis of variance (ANOVA) was performed using SPSS (Statistical Product and Service Solutions, version number is 20.0) software (https://www.ibm.com/products/spss-statistics). Regression analysis on the residual rate of litter and decomposition time and Pearson correlation analysis were performed on each relevant indicator, and the decomposition coefficient *k* was estimated. Excel software was used for data processing and plotting^13,14^.

Mass Retention (*MR*, %) was calculated as

1 $$MR = M_{t} /M_{0} \times {1}00\%$$

where M_0_ is the initial mass of litter (g), and M_t_ is the residual mass of litter after time t (g).

The decomposition dynamic process of litter was described using the Olson exponential decay model:

2 $$M_{t} /M_{0} = a{\mathrm{e}}^{ - kt}$$

In the formula, *a* is the correction coefficient, *k* is the decomposition coefficient of litter, and *t* is the decomposition time, usually represented by a.

The calculation formulas for the time required for 50% decomposition of litter (*t*_*0.5*_) and 95% decomposition time (*t*_*0.95*_) are:

3 $$t_{{0.{5}}} = {\mathrm{ln}}0.{5}/( - k), \;t_{{0.{95}}} = {\mathrm{ln}}0.0{5}/( - k)$$

The formula for calculating the residual decomposition rate (NR, %) of various elements in litter during the decomposition process is:

4 $$NR = M_{t} X_{t} /M_{0} X_{0} \times { 1}00\%$$

where X_0_ is the initial concentration of a certain element in the litter (mg kg^−1^), and X_t_ is the concentration of a certain element in the litter (mg kg^−1^) after decomposition time t. A value of NR < 100% indicates that the element is in a net release state during the decomposition process; NR > 100% indicates a net enrichment state.

## Results and discussion

### Dynamic changes in the decomposition of three types of litter

**Fig. 1:**
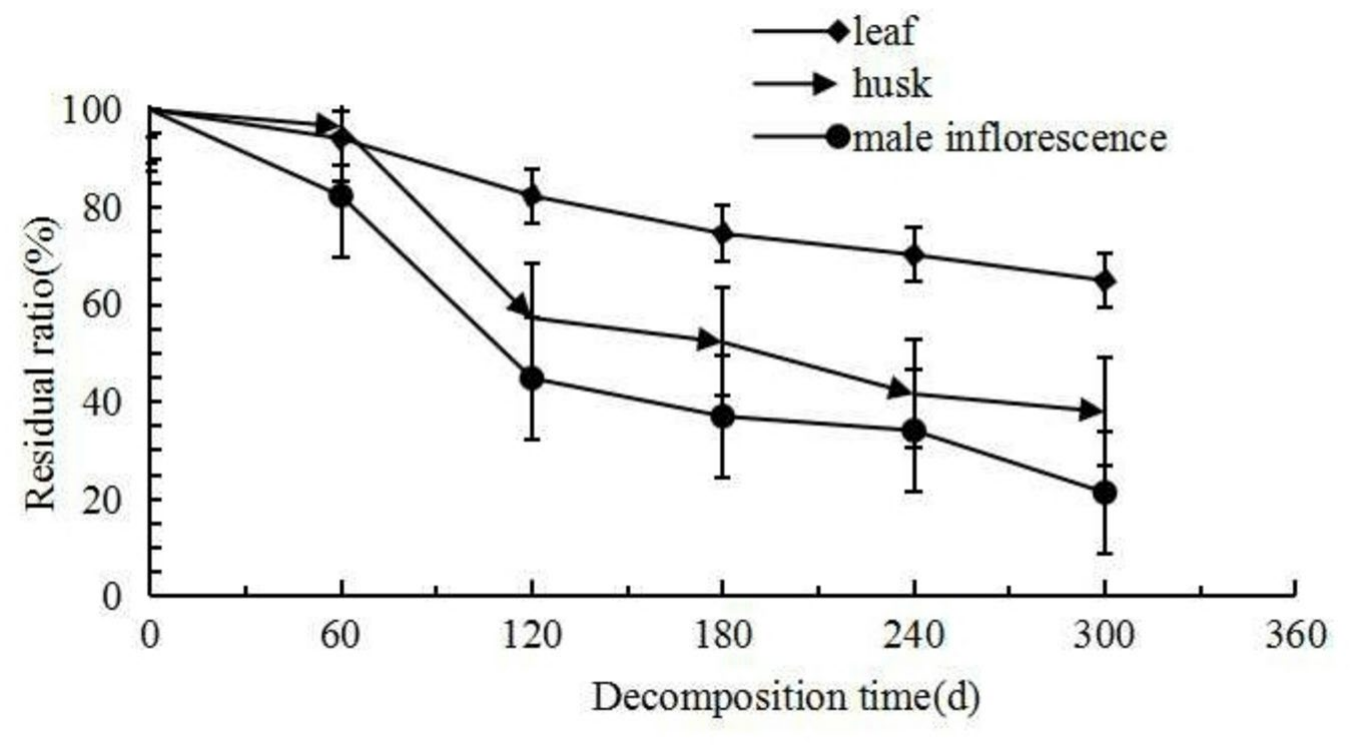
Changes in residual decomposition rates of three types of litter.

The changes in the quality and residual rate of decomposition residues of three types of litter are illustrated in Fig. 1. There were significant differences in the decomposition times between husks and the leaves and male inflorescences. The difference between leaf litter and male inflorescences was not significant. There were significant differences in residual rate between male inflorescences and leaves and husks at the five decomposition points, while the differences between leaves and husks were not significant. The residual decomposition rates of the three types of litter showed a gradually decreasing trend. During the 300-day decomposition process, the dynamic trends of the three types of litter showed a common pattern, i.e., from 0 to 120 days, the residue rate decreased the most, with leaves at 18.67%, husks at 43.79%, and male inflorescences at 55.17%. During the period from 120 to 300 days, the decomposition rate slowed significantly, with decreases of 17.41% for leaves, 19.31% for husks, and 23.50% for male inflorescences. The 120th day marked a turning point in the decomposition process. Before the inflection point, the decomposition process was relatively rapid; after reaching the inflection point, the decomposition curve tended to flatten.

### The decomposition process and model predictions for three types of litter

The correlation and regression results for the decomposition models of the three types of litter are listed in Table 1. The Olson decomposition model had a good fit and met the model requirements. The relationship between the decomposition coefficients k of three types of litter was in the order male inflorescences (1.7852) > husks (1.3140) > leaves (0.5412). The regression model was used to predict the future decomposition process. The estimated times required for 50% decomposition of leaves, husks, and male inflorescences were 1.28a, 0.53a, and 0.39a, respectively, while 95% decomposition would require 5.54a, 2.28a, and 1.68a, respectively.

**Table 1:** Olson decomposition model regression equations for three types of litter.

Litter type	Regression equation	Decomposition coefficient (*k*)	Correlation coefficient (*R*^*2*^)	Half decomposition time (*t*_0.5_) /a	Decompose 95% of time (*t*_0.95_) /a
Leaf	*y* = 1.0037e^−0.5412*t*^	0.5412	0.977	1.28	5.54
Husk	*y* = 1.0395 e^−1.3140*t*^	1.3140	0.895	0.53	2.28
Male inflorescence	*y* = 0.9706 e^−1.7852*t*^	1.7852	0.921	0.39	1.68

y is the residual decomposition rate, and t is the decomposition time.

### Release characteristics of mineral elements

**Fig. 2:**
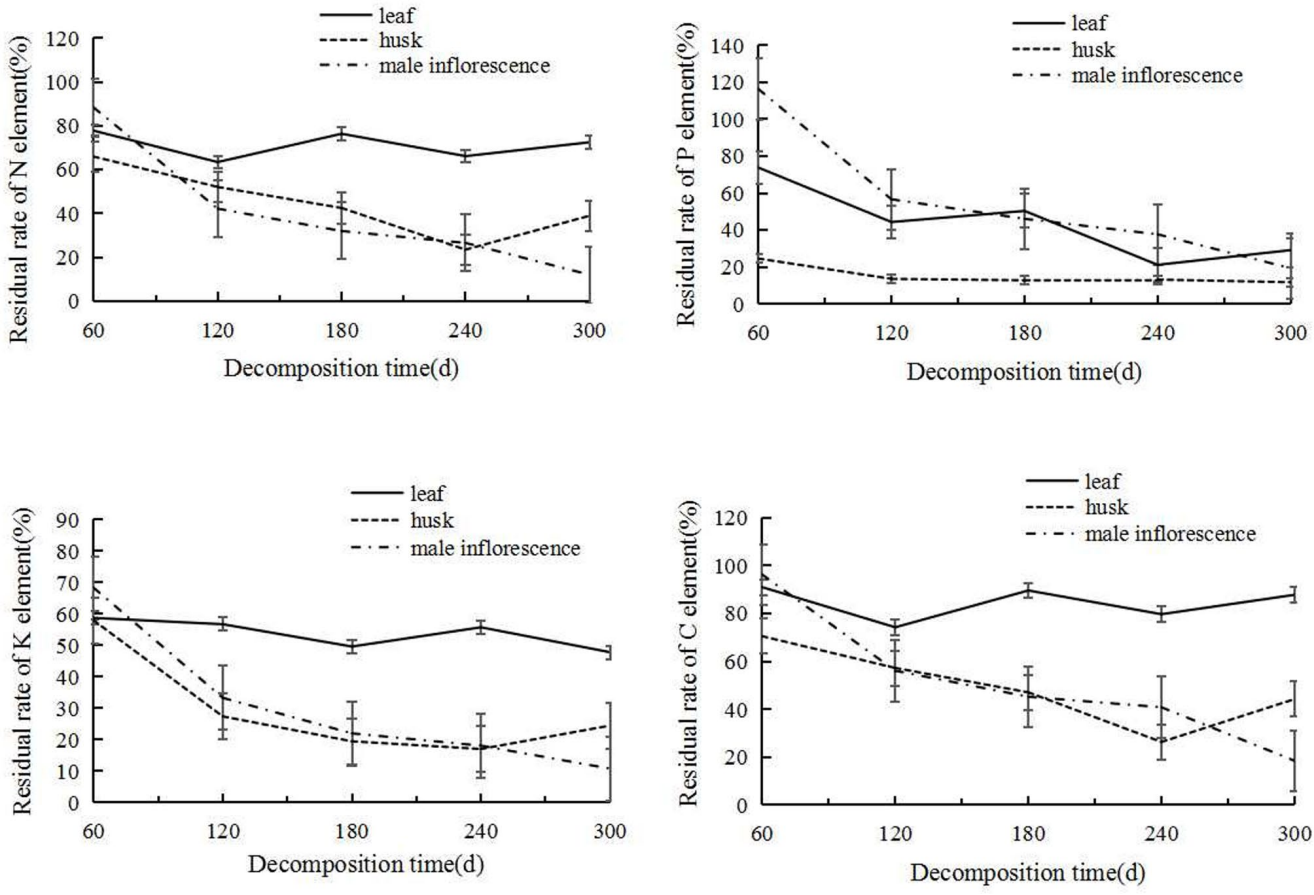
Changes in residual decomposition rates of C N P K element in three types of litter.

The decomposition and release of four mineral elements in the three types of litter were relatively complex, showing different patterns of change (Fig. 2).

The residual rate of leaf-type carbon (C) after 60 days was 90.98%, and the residual rate after 300 days was 87.79%. The intermediate decomposition process was relatively stable, with small fluctuations. The overall change trends of husks and male inflorescences were element release. The male inflorescences continued to release C from 96.24 to 18.50%, while husks showed a continuous release of C before 240 days, from 70.50 to 26.34%. However, C enrichment occurred thereafter, and the residual rate increased to 44.24% after 300 days.

For nitrogen (N), the leaf litter had a similar trend as for C, with a slight fluctuation in the residual rate from 77.71% at 60 days to 72.43% at 300 days, with a slight fluctuating trend during the process. Husk litter continued to release N from 65.88% at 60 days to 23.40% at 240 days, and then began to show element enrichment, reaching 38.88% at 300 days. The male inflorescence litter showed a sustained and stable state of N release, from 88.25% to 11.93%.

For phosphorus (P), leaf litter exhibited a pattern of P release, with the residual rates decreasing from 73.79% at 60 days to 29.17% at 300 days, with a slight fluctuation in the intermediate times. During the decomposition period of 60–120 days, the residual rate of husk litter decreased from 24.60 to 13.62%. From then until 300 days, the residual rate remained stable with little fluctuation and finally reached 11.80%. During the period of 0–60 days, the male inflorescence litter experienced net enrichment of P, increasing from 100 to 116.25%, and then began to release during 60–120 days, with the largest decrease reaching 59.6%. This was followed by a slightly decreasing trend.

For potassium (K), the trend for leaf litter is similar to those for C and N, and the residual rate decreased from 58.71 to 47.81%. The changes in husks and male inflorescences were similar, and the changes in C were also similar. The male inflorescences continued to release K from 68.26 to 10.79%; husks also showed continuous release before 240 days, decreasing from 57.90 to 17.03%, and then showed a slight enrichment, with a final residual rate of 24.46%.

### Release characteristics of trace elements and lignin

**Fig. 3:**
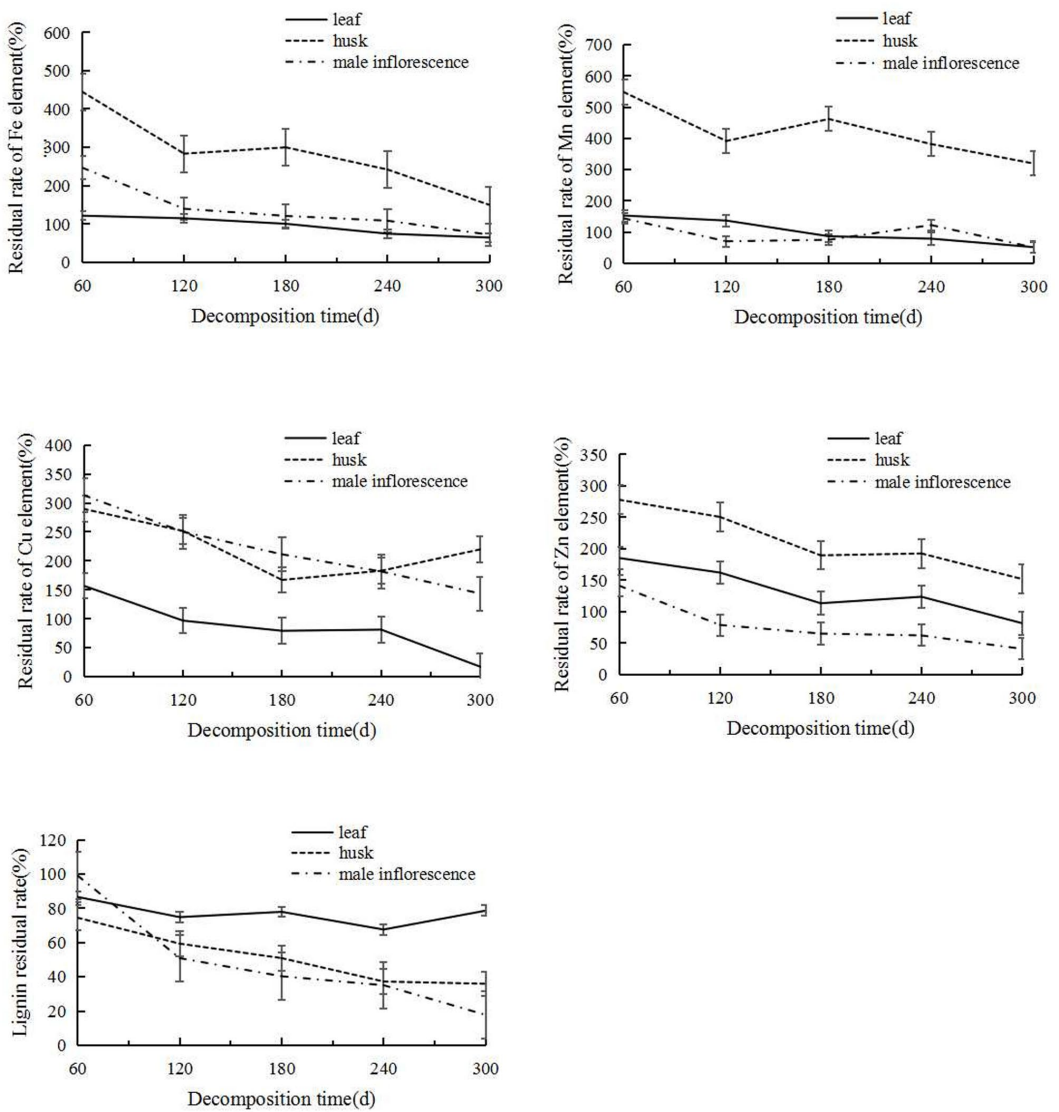
Changes in residual decomposition rates of Fe Cu Zn Mn and Lignin elements in three types of litter.

The release of various trace elements and lignin varied among the three types of litter (Fig. 3). Overall, all three types of litter showed a continuous decrease in residual rates of Fe, as Fe continued to be released. We note that Fe underwent different degrees of net enrichment during the decomposition process. The leaves showed a net release during 0–180 days, with a maximum of 121.54% and a minimum of 100.36%. The husk litter showed a net enrichment throughout the entire period, with a maximum of 444.79% and a minimum of 149.70%. The male inflorescences reached a maximum enrichment value of 246.75% at 60 days, and then the percentage gradually decreased, with a net release state of 71.91% at 300 days.

The overall trend of Mn in leaf litter was “enrichment release,” with a net enrichment before 120 days and a net release thereafter. The husk litter exhibited a net enrichment state throughout the entire decomposition period, but the rate showed an alternating pattern. The pattern of variation in male inflorescence litter was similar to that of husks, but the enrichment peak was only 143.98%, and there were three net release values during the process; the value at 300 days was 50.66%.

Copper (Cu) showed an overall pattern of enrichment release in leaf litter, reaching a maximum enrichment value of 157.00% at 60 days, and Cu continued to be released, reaching 17.09%. The husk litter showed a net enrichment during the entire decomposition period, but the rate showed a trend of rising–falling–rising, with a maximum residual rate of 289.71% and a minimum of 167.09%. The overall decomposition of male inflorescences showed a net enrichment, reaching a peak of 313.47% at 60 days. Afterward, the enrichment rate continued to decrease, finally reaching 143.50%.

For Zn, the decomposition and release trends of the three types were basically the same, and the residual rate showed a stepwise downward trend. The leaf litter showed net enrichment before 240 days, and the release rate exceeded the enrichment rate thereafter, reaching the lowest value of 81.45% at 300 days. Husk litter Zn decreased from 277.64% at 60 days to 151.62% at 300 days. The male inflorescences exhibited net enrichment from 0 to 60 days, followed by a continuous increase in the release rate, reaching the lowest value of 40.71% at 300 days.

Lignin showed a net release state at all stages. The leaf litter lignin decreased from 86.62% to 78.61%, with a relatively small overall change and a trend toward a stable release state. The husk litter showed a continuous release state, and the residual rate decreased from 74.47% to 35.94%. Within the first 120 days, the residual rate of male inflorescence litter varied by 49.10%, and within the 180 days thereafter, the variation was 33.14%, with a gradually slowing release rate.

**Fig. 4:**
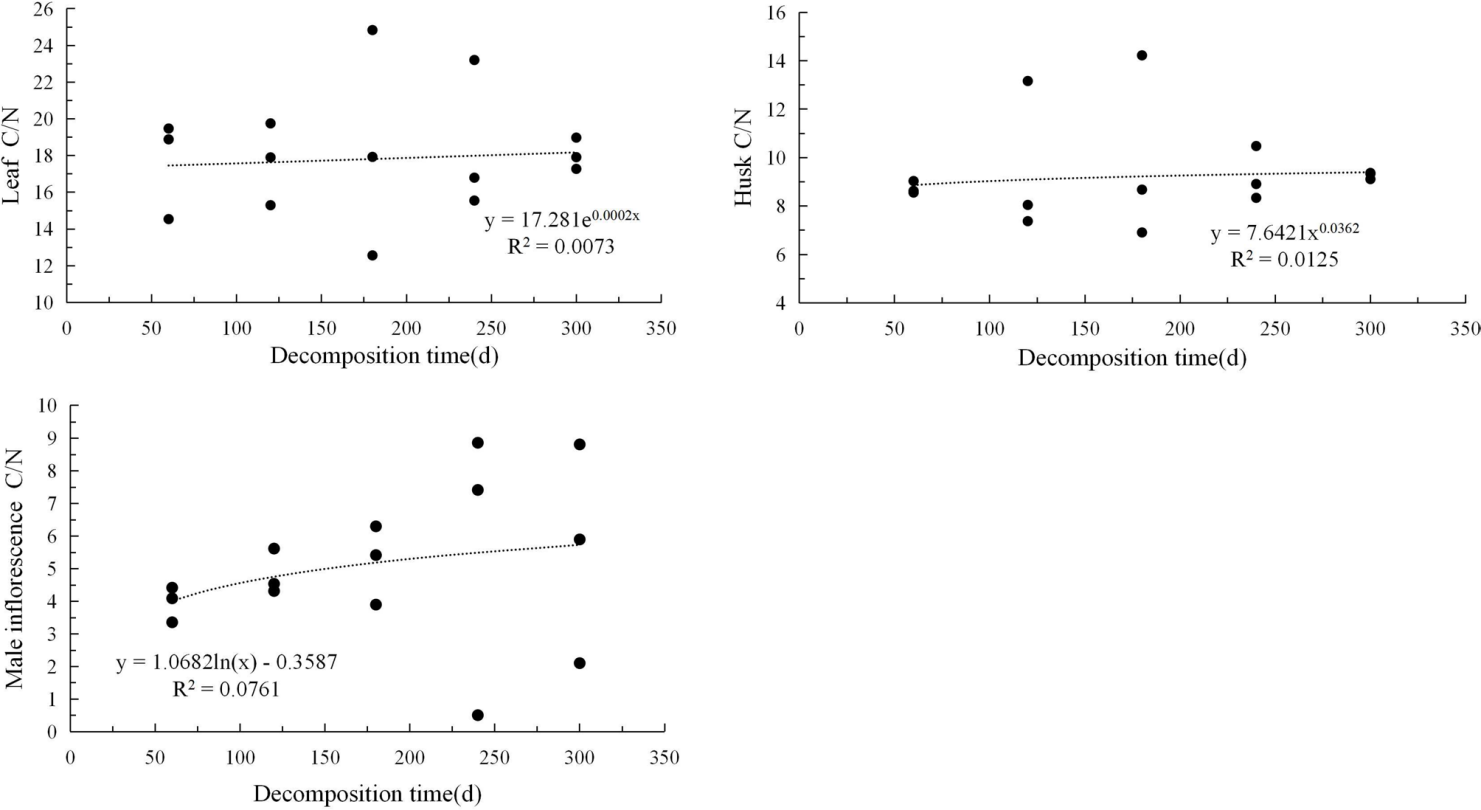
Changes in carbon to nitrogen ratio in three types of litter.

**Fig. 5:**
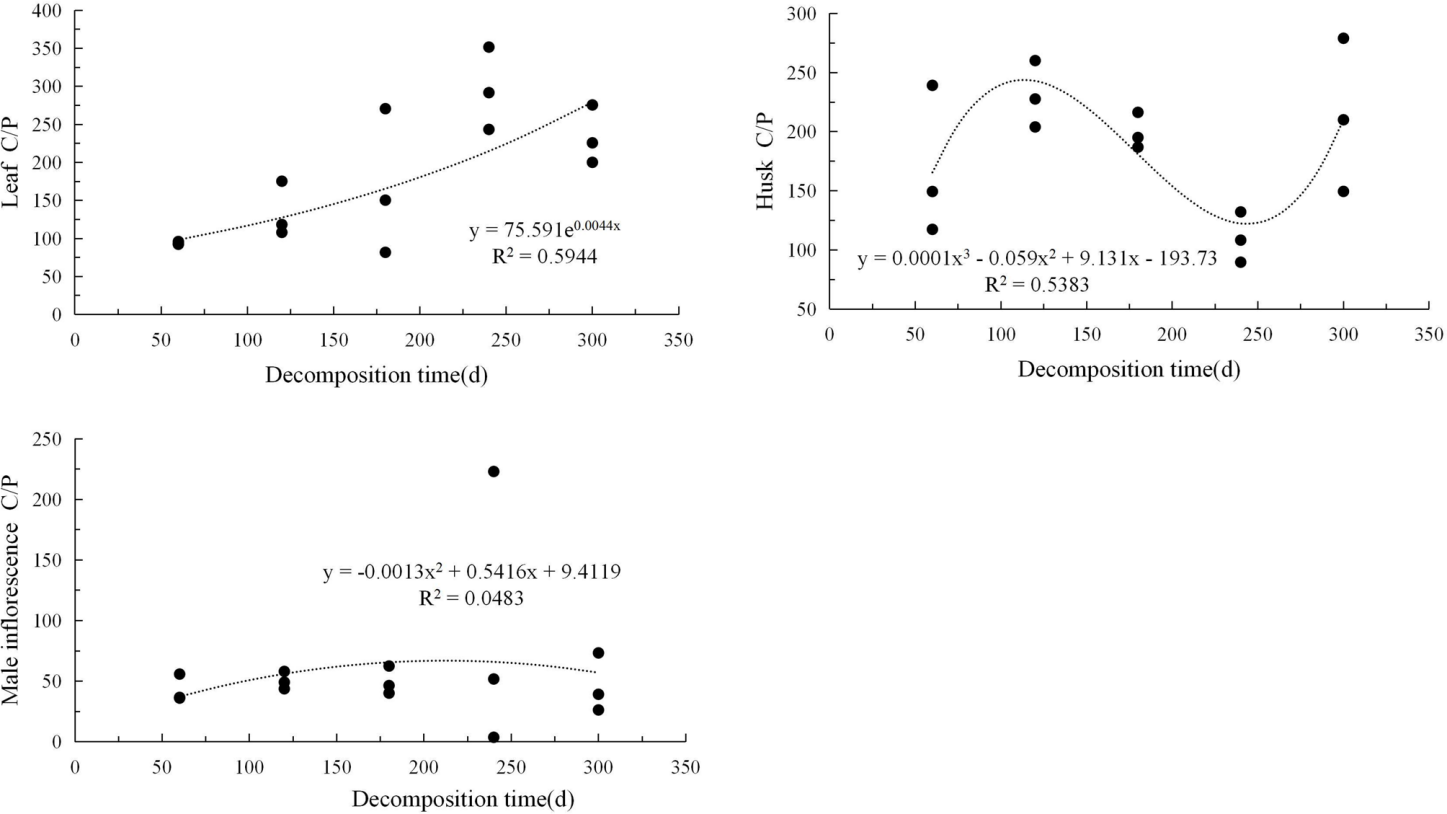
Changes in carbon phosphorus ratio in three types of litter.

**Fig. 6:**
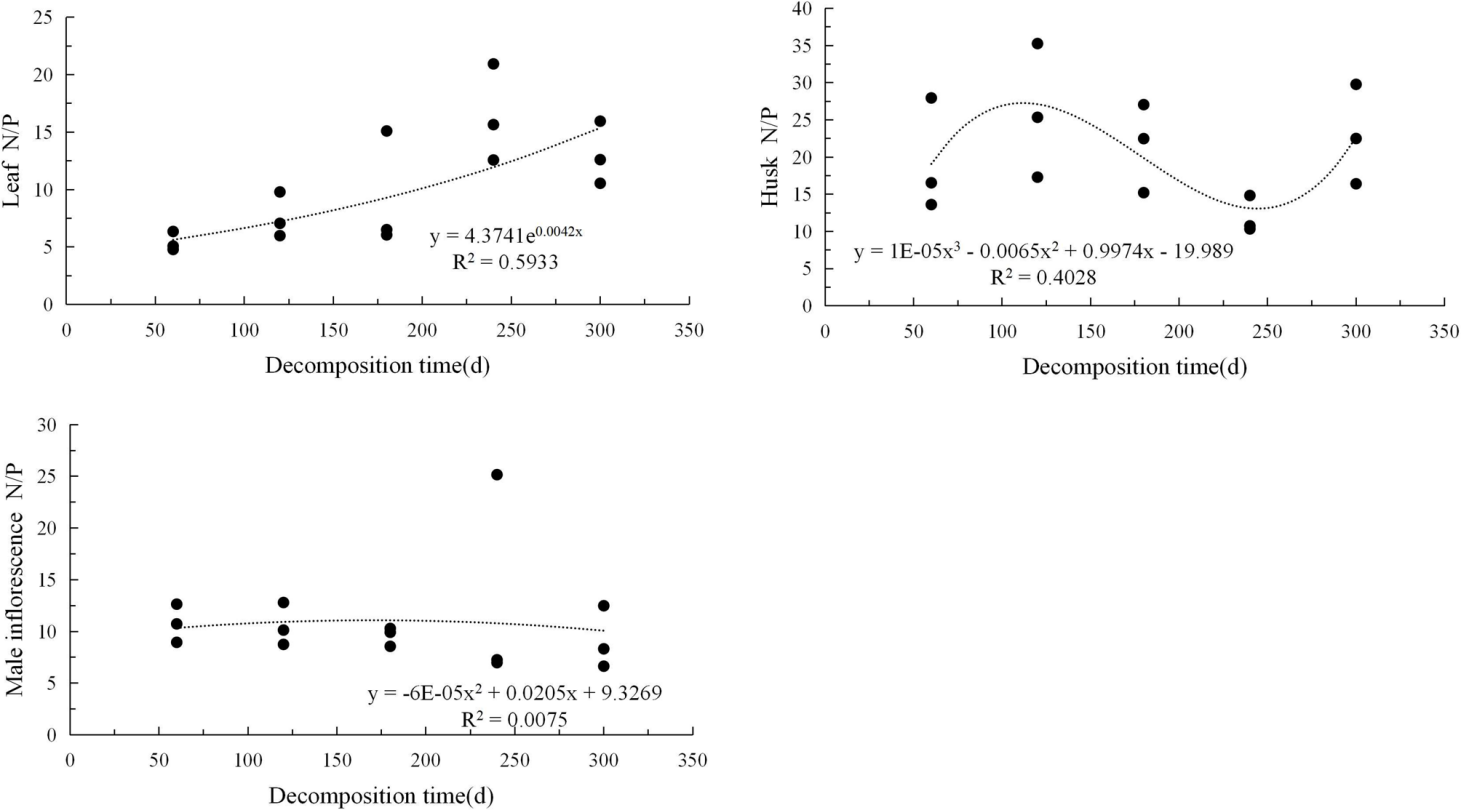
Changes in nitrogen phosphorus ratio in three types of litter.

The carbon-to-nitrogen ratio, the carbon-to-phosphorus ratio, and the nitrogen-to-phosphorus ratio of the three types of litter exhibited different trends and patterns with decomposition time (Figs. 4, 5, 6). The correlation between C/N and decomposition time for the three types of litter could be described using an exponential model and showed a continuous upward trend during the decomposition period. The C/P situation was relatively complex, and the leaf litter showed an exponential upward trend; the husk litter showed a cubic regression relationship, with the carbon-to-phosphorus ratio increasing initially, then decreasing, and then increasing again, showing a clear fluctuation trend with peaks and troughs. The male inflorescence litter followed a quadratic regression relationship, with no significant fluctuation trend and overall showing an upward and then downward trend. The N/P variation shows an exponential upward trend for leaf types, a trinomial regression relationship for husk litter, and a linear downward trend for male inflorescence types.

### Analysis of the factors affecting the decomposition coefficients (K) of three types of litter

As shown in Table 2, the decomposition coefficient k was positively correlated with the decomposition time, nitrogen content, carbon content, lignin content, and C/P, while negatively correlated with litter residue, phosphorus content, C/N, and lignin/nitrogen. The coefficient was significantly positively correlated with litter residue (*p* < 0.05).

**Table 2:** Correlation analysis between decomposition coefficient k and various influencing factors.

Decomposition coefficient	Decomposition time	Residual amount of litter	Nitrogen element	Phosphorus element	Carbon	lignin	C/N	C/P	Lignin/Nitrogen
*k*	0.990	− 0.761*	0.642	− 0.562	0.644	0.740	− 0.848	0.980	− 0.464

* indicates a significant correlation at the 0.05 level.

## Discussion

Litter decomposition is a relatively complex physical and chemical process that is influenced and constrained by various factors^15^. The data from this study show that after 10 months of decomposition under natural conditions, the residual mass proportions of the three types of litter, from high to low, were leaves (64.92%) > husks (37.90%) > male inflorescences (21.33%). The decomposition degree of husks and male inflorescences exceeded 60%. This result is related to two factors: the chemical properties of the three types of litter in their initial state and the environmental conditions of the sample area, with moderate sunlight, mild temperature and humidity changes, and adequate microbial activity. It should be noted that the decomposition dynamics of the three types of litter showed a common pattern, i.e., from 0 to 120 days, the residual proportions of the litter rapidly decreased, while the decomposition rate gradually increased; from 120 to 300 days, the residual rate slowly decreased; the decomposition rate gradually decreased, and the decomposition process tended to stabilize. At 120 days, there was an inflection point in the decomposition process; this was consistent with previous research results. The reason for the result was that the experimental decomposition started in May of that year, and the 120th day was in September. This period is the active period for tree development and growth, with frequent exchange of nutrients above and below ground. Microbial activity is the highest at this time of the year, and thus the rate of decomposition continued to increase, reaching a peak at around the 120th day. Subsequently, changes in the environmental conditions not conducive to decomposition gradually increased, and the decomposition rate slowed.

The decomposition of litter is a dynamic process, and most researchers use mathematical models to describe and predict the decomposition process. In terms of the fitting effect of model regression, the Olson model is the current optimal choice. Specifically, in this study, the correlation coefficients of the Olson regression equation results for leaves, husks, and male inflorescences were 0.977, 0.895, and 0.921, respectively, indicating a high degree of fit. This model can be used for both simulation and prediction. The 50% decomposition of leaves, husks, and male inflorescences required 1.28a, 0.53a, and 0.39a, respectively, while the 95% decomposition required 5.54a, 2.28a, and 1.68a, respectively. Table 3 lists the region where this study was located and the seven geographical regions cover the Northeast, Northwest, North China, South China, Southwest, and Southeast regions nationwide, reflecting the Olson model prediction data of multiple tree species and litter types under various climatic conditions. The data in the table also indicate a trend where the decomposition process is constrained, and the decomposition cycle is long in regions with relatively prominent climate and environmental factors (such as the Northeast and Southwest regions). In areas with relatively average environmental factors, microbial activity is high; decomposition is relatively rapid, and the decomposition cycle is short, as in the husks and male inflorescences in this study. The data also confirm the conclusion that different types of litter components from the same tree species have different decomposition rates^21^.

**Table 3:** Comparison of data between this study and other location studies in China.

Geographical location	Varieties of trees	Litter type	Decompose 50% of time (a)	Decomposition 95% time (a)	Data sources
Nanjing City, Jiangsu Province	*Populus deltoides Bartr. cv*	leaf	0.65	2.92	^15^
Garze Prefecture, Sichuan Province	*Abies fabri*	leaf	3.69	15.93	^16^
	*Rhododendron williamsianum*	leaf	2.60	11.22	
Ulanqab City, Inner Mongolia Autonomous Region	*Stipa breviflora*	Branch and leaf mixture	2.09	10.05	^17^
Sanming City, Fujian Province	*Castanopsis carlesii* (Hemsl.) Hay	Blade mixture	0.65	2.81	^18^
	*Liriodendron chinense* (Hemsl.) Sargent		1.05	4.55	
The Greater Khingan Mountains region of Heilongjiang Province	*Larix gmelinii* (Rupr.) Kuzen	Branch and leaf mixture	4.78	20.66	^19^
Fangchenggang City, Guangxi Zhuang Autonomous Region	*Ficus altissima*	A mixture of branches, leaves, flowers, and fruits	0.42	1.83	^20^
Xingtai City, Hebei Province	*Juglans regia L*	leaf	1.28	5.54	This study
		husk	0.53	2.28	
		male inflorescence	0.39	1.68	

The return of litter is still an important source of soil organic carbon accumulation and an important ecological process for maintaining the above ground and underground carbon pools in the symbiotic system between plants and soil. During the decomposition process of different types of litter, various macroelements, trace elements, and metal elements re-enter the aboveground underground nutrient cycling system. Understanding the decomposition characteristics of different types of litter is of fundamental significance for optimizing daily nutrient management and continuously restoring and improving soil fertility in walnut orchards. The decomposition process of litter is accompanied by the release or enrichment of nutrients such as C, N, P, and K. These processes are significant for nutrient cycling in artificial forest ecosystems^22–24^. In this study, the decomposition and release of four mineral elements in the three types of litter were relatively complex and did not show consistent patterns of change. Existing research results indicate that the release of nutrients from litter generally manifests as three types: the direct release mode, the enrichment release mode, and the leaching enrichment release mode. In this study, the release characteristics of C and N elements in the three types of litter were consistent. The leaf litter showed a leaching enrichment release pattern for C and N, while the husk litter showed a slightly different release enrichment pattern. The male inflorescences showed a direct release pattern, consistent with the results of other researchers^25–27^. As the main component of organic matter in litter, C does not show a stable and continuous release during the decomposition process. In the early stages of decomposition, there is a continuous loss by leaching; as this process weakens, C appears to be enriched and then is continuously released. The N element exhibits two release characteristics in both the husk and male inflorescence litter that may be directly related to the initial N content of the two types of litter. The initial N content in leaves was significantly lower than that in the husks and male inflorescences types (Table 4), making it difficult to meet the N demand of microorganisms for decomposition activity. Microorganisms absorb N from the environment for transformation, leading to enrichment. The initial N content in husks and male inflorescences was relatively high, sufficient to meet the requirements for microbial decomposition. N is released as the decomposition process continues. Compared with other nutrient elements, the leaching of P displays a higher release rate in the early stages. When the decomposition reaches a certain level, the release rate significantly slows and tends to a stable value. The data from this study confirm this pattern^28,29^.

**Table 4:** Initial chemical properties of three types of litter.

Indicator name	Leaf	Husk	Male inflorescence
N content (g kg^−1^)	31.360 ± 0.69b	40.402 ± 2.66a	55.220 ± 4.87a
P content (g kg^−1^)	6.170 ± 0.11a	6.069 ± 0.11a	3.960 ± 0.81b
K content (mg kg^−1^)	817.558 ± 23.81b	1332.552 ± 136.38a	878.046 ± 11.58ab
C content (g kg^−1^)	466.302 ± 9.26a	329.403 ± 16.41b	200.324 ± 10.68c
Fe content (mg kg^−1^)	731.746 ± 27.39a	181.292 ± 12.12c	326.329 ± 38.03b
Mn content (mg kg^−1^)	105.829 ± 20.17a	11.976 ± 3.30b	62.330 ± 23.02a
Cu content (mg kg^−1^)	52.751 ± 12.26a	5.000 ± 3.27b	7.879 ± 2.94b
Zn content (mg kg^−1^)	28.983 ± 10.71a	8.000 ± 3.27b	46.110 ± 5.35a
lignin content (g kg^−1^)	329.946 ± 15.38a	346.093 ± 10.92a	32.123 ± 1.72b
C/N ratio	14.869 ± 0.50a	8.153 ± 0.92b	3.628 ± 0.43c
C/P ratio	75.574 ± 1.87a	54.280 ± 3.05b	50.581 ± 8.66b
N/P ratio	5.083 ± 0.20b	6.658 ± 0.46b	13.943 ± 3.99a

The data in the table is the average value, and different letters in the same row indicate significant differences (P < 0.05).

The return of trace elements from litter to the soil is an important pathway for nutrient cycling in ecosystems. The release/enrichment characteristics of trace elements are an important way to measure their ability to return elements and are a key process in promoting and maintaining soil plant element cycling^30,31^. In this study, during a 10-month decomposition cycle, the four trace elements of the three types of litter showed an overall release pattern, consistent with many existing research results^32–34^. However, there were some exceptions, such as the residual rate of Fe in husks at 300 days being 149.70%, and the residual rate of Mn in husks at 300 days being 319.68%. Although there was an enrichment phenomenon, the change trend indicated that if the decomposition cycle is extended, the overall residual rate still showed a continuous decrease, while the element content showed a release trend. During the decomposition of litter, most of these trace elements can combine with humic substances and form relatively stable complexes^35^, or they can be fixed by microbial decomposition activity^36^. As decomposition proceeds, these substances are ultimately released through mineralization, and these intermediate links lead to enrichment or fluctuating release during the decomposition process^36^.

Lignin degradation is carried out under the combined effects of litter substrate quality, soil biological activities, and abiotic factors^37^. In this study, lignin in the three types of litter showed relatively stable release characteristics. Lignin is a substance that is difficult for microorganisms to decompose, and thus a high lignin content will inhibit decomposition^38,39^. The initial lignin content of male inflorescences was the lowest, and the decomposition rate was the highest; the initial content of husks was greater than that of leaves, although the difference was not significant, but the decomposition rate of husk type was significantly higher than that of leaves. This may be because lignin was not a key determinant of litter under the specific environmental conditions of this study, and the final result was also constrained by other factors. The fluctuating enrichment phenomenon that occurred during the decomposition process was because microorganisms can form lignin analogs through decomposition activities, resulting in lignin enrichment. The degradation of lignin is carried out by fungi and bacteria, and the number and activity of microbial decomposers directly affect the degradation process^40^.

Different parts of plants have different decomposition rates due to their properties. The study on the decomposition rate of root, stem, and leaf litter of five grassland plants found that the decomposition rate of stems and leaves was significantly positively correlated with their initial N and P content, while the decomposition rate of roots was significantly negatively correlated with the initial C/N ratio^41^. Related research has shown that under the same environmental conditions, the decomposition rate of litter is closely related to its initial chemical properties. Generally, low-quality litter decomposes faster, while high-quality litter is more difficult to decompose^42^. Litter has a higher level of C components (such as lignin) or a higher C/N and C/P ratio, usually manifested as a slow decomposition rate and a long decomposition cycle. If the N/P ratio is high, decomposition proceeds more rapidly^43,44^. As shown in Table 4, the initial N contents were significantly higher in male inflorescences and husks than in leaves, while the C/N ratio showed an opposite relationship. The decomposition rate was in the order of male inflorescences > husks > leaves. This result confirms a widely recognized rule by most researchers, i.e., the quality of litter substrate is the best predictor of the decomposition coefficient. The initial N content of litter and the C/kN ratio are significantly correlated with litter decomposition, with a higher N content; the lower the C/N ratio, the faster the decomposition of litter^45,46^.

The decomposition process of litter is influenced by both external environmental factors and internal component factors^47^. External environmental factors include temperature, humidity, rainfall, wind speed, soil structure, and microbial species and activity^48^, while internal component elements refer to initial chemical and physical properties. In this study, the correlation between initial chemical properties and decomposition rate was explored. The analysis results confirmed that the higher the N content and the lower the C/N ratio, the greater the decomposition rate. This pattern has been repeatedly confirmed in many studies^12,15,19,22,26,27^.

## Conclusion

1. During the 300-day decomposition period, the mass residues of the three types of litter ranked from high to low were as follows: leaves (64.92%) > husks (37.90%) > male inflorescences (21.33%). The 120th day was a turning point in the decomposition process. Before the turning point, the decomposition process was active and rapid; after reaching the inflection point, the rate of decomposition tended to slow.
2. The Olson model was used to fit and predict the decomposition process of three types of litter. The correlation analysis indicated a high fitting degree. The estimates of the decomposition coefficient *k* of litter were as follows: male inflorescences (1.7852) > husks (1.3140) > leaves (0.5412). The higher the *k* value, the faster the decomposition. The decomposition of 50% of leaves, husks, and male inflorescences required 1.28a, 0.53a, and 0.39a, respectively, while the 95% decomposition required 5.54a, 2.28a, and 1.68a, respectively.
3. After 300 days of decomposition, according to the criterion of NR < 100%, the C, N, P, K, and lignin contents of the three types of litter all showed a net release, with a fluctuating pattern and a slight enrichment phenomenon occurring during the process. Compared with the initial state of decomposition, the four trace elements generally showed a net release pattern.
4. Based on Pearson correlations with three types of litter decomposition indices, the decomposition coefficient k was positively correlated with the decomposition time, nitrogen content, carbon content, lignin content, and C/P ratio while being negatively correlated with litter residue, the phosphorus content, the C/N ratio, and the lignin/nitrogen ratio, consistent with most research results.
5. In future research, more attention should be paid to the element return, absorption, and decomposition processes and driving factors of the plant litter soil ecological complex, providing reference for nutrient management and soil fertility improvement in walnut orchards.

## Data Availability

All data generated or analysed during this study are included in this published article.
